# The Reliability and Validity of Using Ice to Measure Cold Pain Threshold

**DOI:** 10.1155/2017/7640649

**Published:** 2017-08-02

**Authors:** Prue Tilley, Leanne Bisset

**Affiliations:** ^1^Menzies Health Institute Queensland, Griffith University, Gold Coast, QLD, Australia; ^2^Allied Health Services, Logan Hospital, Metro South Health, Logan, QLD, Australia

## Abstract

Cold pain threshold (CPT) measures an individual's pain threshold in response to a cold stimulus. CPT is most accurately determined with specialised equipment; however this technology is not readily accessible to clinicians. Instead, ice has been employed to measure CPT. An optimal ice protocol has not yet been identified. The aim of this study was to evaluate the reliability and validity of two CPT protocols using ice in a young, healthy population. Twenty-two participants aged 22.6 (SD 1.81) years underwent CPT measurements over 6 anatomical sites across 3 protocols, which were repeated in 2 sessions. One protocol measured pain (PVAS) following ice applied for a standardised period of 30 seconds; a second protocol measured time to onset of pain, and the reference standard measured CPT using laboratory equipment (TSA-II). The PVAS protocol demonstrated the best reliability (mean ICC 0.783, 95% CI 0.706 to 0.841), but the Timed protocol demonstrated superior validity compared to the reference standard (mean ICC −0.504, 95% CI −0.621 to −0.365).

## 1. Introduction

It is well established that an individual's hyperalgesic response to a cold stimulus may be indicative of alterations in somatosensory processing [1]. Cold pain threshold (CPT) is used to measure cold hyperalgesia and is defined as the temperature at which a sensation of cold changes to a sensation of cold-with-pain. In cold hyperalgesia, a patient's CPT is reached at a significantly warmer temperature than a healthy individual's CPT. Widespread cold hyperalgesia has been associated with chronic pain conditions such as lateral epicondylalgia, whiplash associated disorders, chronic low back pain, fibromyalgia, osteoarthritis, and rheumatoid arthritis [2–6]. In addition, cold hyperalgesia has been associated with a more severe initial presentation and is predictive of prolonged recovery time and poorer long-term outcomes [3, 5, 7]. Cold hyperalgesia is considered a feature of central sensitisation, which may require alternative treatments as patients may not respond to traditional approaches [3].

Quantitative sensory testing (QST) is used in a laboratory setting to quantify a person's threshold of sensation by applying a stimulus to the skin and comparing the results to either the individual's unaffected side or to normative population values [8]. Determining CPT in a clinical setting may provide the clinician with insight into the underlying pain processing mechanisms, provide an alternative means by which the progression of the condition could be monitored, or be used as an outcome measure to assess the effects of targeted treatment interventions [9]. Despite the potential clinical value in CPT testing, there is presently little evidence of a reliable and valid method of assessing this in a clinical setting without the use of expensive equipment. This prohibitive cost of gold standard QST equipment renders the QST technology inaccessible to the majority of health clinicians. Previous research has suggested two different methods for CPT determination in a clinical environment using ice. Cathcart and Pritchard [10] used ice that was applied to the skin and the time taken to reach the participant's CPT (described as the point where discomfort is reached) was recorded. Maxwell and Sterling [11] applied ice to the skin for a standardised period of time (30 seconds) and participants rated their discomfort level on a visual analogue scale. The reliability and validity of these two testing protocols have not yet been determined in comparison to a proven measure such as the TSA-II. Ice has been proposed as a cheap and easily accessible solution to use in the assessment of CPT. However, at this stage a standardised protocol has not yet been developed. Therefore, the aim of this study was to determine the validity and test-retest reliability of two CPT protocols using ice, in a healthy control population. A second aim was to use limits of agreement (LoA) to indicate how large a difference in CPT measures was necessary to achieve 95% confidence of a real difference in CPT for each protocol.

## 2. Materials and Methods

This study employed a single cohort repeated measures cross-over design. Twenty-two (12 women) healthy participants from a university community were recruited, with a mean age of 22.6 (SD 1.81) years. Participants were screened to ensure they had been free of musculoskeletal injuries or any pain condition for the previous two months and had no history of neurological injury or disease. Written informed consent was obtained from each participant prior to data collection and the study was approved by the University's Human Research Ethics committee. In the 24 hours prior to testing, participants were advised to avoid medications such as analgesics, which may alter sensory perception, and to avoid the use of moisturisers or body oils. Demographic data including age, height, weight, and past history of injury were recorded on the first day prior to testing.

## 3. Procedure

All tests were carried out in a laboratory setting between 9 am and 1 pm in June 2014. Participants were tested in two sessions separated by 48 hours and were given standardised instructions prior to each test. There were three isolated testing rooms, each designated to one test protocol, with a controlled temperature of 24°C. The three different test protocols were each performed by a different assessor who was blind to the results of the other protocols. The protocols were administered in a randomised order within a single session and were repeated in a 2nd session approximately 48 hours later. The same assessors administered each protocol between sessions in an effort to ensure consistency across the test sessions. The order of protocol testing was randomised between sessions, as was the starting anatomical location for each test protocol.

Six anatomical locations were chosen for testing, based on clinically relevant chronic musculoskeletal injury sites with which central sensitisation is commonly associated. Unilateral testing was performed on the participant's dominant side, which was determined via handedness, as previous work has found no significant difference between left and right limbs [8]. Participants lay supine on a treatment bed and each anatomical location was identified by palpation and the skin was marked with a water-soluble pen (Table 1), to ensure the consistent placement of stimuli across the three protocols. Each stimulus was applied to anatomical locations in the order listed in Table 1; however the starting location was randomly assigned to each participant in each protocol. Testing at each anatomical location was performed three times for each protocol, and the mean values were used in further analyses. Participants were given a 10-minute break between protocols to minimise the likelihood of temporal summation occurring from repeated stimuli. Environmental conditions and instructions were consistent for all participants.

## 4. Protocols

The two ice protocols examined in this study will be termed the “Timed” protocol and the “PVAS” (pain visual analogue scale) protocol. The reference standard for measuring CPT was the Thermosensory Analyzer (TSA-II, Medoc, Israel).

### 4.1. Reference Standard

A 4 cm^2^ thermode was directly applied to each marked anatomical location and the initial temperature of the thermode was set at 32°C. Consistent with QST recommendations from the German Research Network on Neuropathic Pain [8], a standard protocol for determining CPT was used. The thermal stimuli decreased at a rate of 1°C per second and participants were instructed to press a hand-held button when the sensation changed from cold to cold-with-pain, at which point the thermode returned to the 32°C baseline value. There was a 30-second break between each application of the stimulus for a total of three repeated measures. The results were digitally recorded within the TSA-II software program and later transferred to an electronic database for further processing.

### 4.2. Timed Ice Protocol

A 30 mL plastic syringe (20 mm diameter), filled with water and frozen, was used in both of the ice protocols. The tip of the syringe was cut off and the ice tube was pushed through the syringe to expose an ice cylinder of approximately 20 mm diameter. For the Timed protocol, the ice was directly applied to an anatomical location and a stopwatch timer was started. The ice stimulus was held in place until the sensation experienced by the participant changed from one of cold to one of cold-with-pain, at which point the participant was asked to say “stop.” The duration of time was manually recorded and the assessor then moved to the next anatomical location. The test was terminated at a maximum of 60 seconds. One measurement of CPT was taken at each anatomical site and the order was repeated until each anatomical site was measured three times.

### 4.3. Pain Visual Analogue Scale (PVAS) Ice Protocol

The 20 mm diameter ice cylinder was directly applied to the marked anatomical site and a stopwatch timer was pressed. The ice stimulus was held in place for 30 seconds, after which the participants rated their pain on a 100 mm pain visual analogue scale, with 0 mm indicative of no pain at all and 100 mm indicative of the worst pain imaginable. This pain score was manually recorded. As per the Timed protocol, each anatomical location was tested once in sequential order and the order was repeated three times.

## 5. Data Analysis

Triplicate recordings from each session at each anatomical location were averaged for analyses. SPSS statistical software (V22, IBM, Chicago USA) was used and significance was set at <0.05. To examine the reliability between the two sessions for each protocol, Intraclass correlation coefficients with 95% confidence intervals (ICC_3,1_) were calculated for each anatomical location, based on an absolute agreement, 2-way random effects model. A repeated measures 2-way analysis of variance (ANOVA) with within-subjects factors of session (2 levels) and anatomical location (6 levels) was used to assess differences between sessions and anatomical locations for each protocol. The effect of the order of testing was assessed by entering it into the model as a covariate, with adjusted results presented if it was found to significantly influence the results. Significant main or interaction effects identified in the ANOVA were further explored via post hoc *t*-tests. If there were no significant main or interaction effects, the average between sessions was calculated and used in further analyses.

To examine the validity of both the Timed ice protocol and the PVAS ice protocol against the reference standard, simple linear regression analyses were calculated to predict each ice protocol (independent variables) against the gold standard (dependent variable) for pooled anatomical locations. Criteria for determining the strength of the correlations were based on the general rules where ICCs below 0.5 represent poor reliability, ICCs from 0.51 to 0.75 indicate moderate reliability, and ICCs over 0.75 represent good reliability [12].

The limits of agreement (LoA) were calculated for each anatomical location within each protocol, to determine the change in measurement that is required in order to be 95% confident that the change is larger than the measurement error [13].

## 6. Results

Overall, there was no significant difference between sessions for any of the protocols (Table 2), except for the Timed protocol (*P* = 0.02), where the time to pain onset at the elbow was different by 4.8 (95% confidence intervals (95% CI) 1.5 to 8.2) seconds between sessions. Given that the LoA calculated for the elbow using the Timed protocol ranged from −10.0 to 19.6 seconds (Table 2), the sessions for each anatomical location were averaged for all protocols and used in further analyses. The order of testing did not significantly influence any of the outcomes (*P* ranged from 0.768 to 0.909). Intraclass correlations (ICC_3,1_) and LoA between the two sessions for each protocol are reported in Table 2. The reliability between sessions was good in the PVAS protocol (mean ICC 0.783, 95% CI 0.706 to 0.841) and moderate in the Timed ice protocol (mean ICC 0.714, 95% CI 0.619 to 0.788).

There was a significant main effect for anatomical location across all protocols (*P* < 0.001). On post hoc testing, the measures recorded at the neck appeared to be consistently more sensitive when compared with other anatomical locations (Table 3). PVAS protocol was significantly associated with the reference standard (*F*(1,130) = 17.685, *P* < 0.001), with an *R*^2^ of 0.12. Participant's predicted CPT is equal to 9.6 + 1.6 (PVAS) °C when PVAS is measured in mm. Participants' CPT increased by 1.6°C for every mm of increase in pain severity.

Similarly, the Timed protocol was significantly associated with the reference standard (*F*(1,130) = 69.171, *P* < 0.001), with an *R*^2^ of 0.35. Participant's predicted CPT is equal to 19.6–0.3 (time) °C when time is measured in seconds. Participants' CPT increased by approximately 1°C for every 3-second increase in time to pain onset (Table 3).

## 7. Discussion

The primary aims of this study were to investigate the reliability of two clinical protocols measuring CPT using ice and compare these protocols to a reference standard (TSA-II) to determine their validity. The PVAS protocol had the strongest reliability between sessions, ranging from 0.569 to 0.870. The Timed protocol had weak to good reliability between sessions, ranging from 0.483 to 0.868. Surprisingly, the reference standard TSA-II exhibited poor to moderate reliability between sessions, ranging from 0.185 to 0.679. In addition, there was poor association across all anatomical locations between TSA-II and PVAS protocols, with ICCs ranging from −0.013 at the neck to 0.214 at the knee. There was weak to moderate negative correlation between TSA-II and Timed protocols (ICCs from −0.254 to −0.530). This was particularly evident in the neck, elbow, and thigh locations. This negative association between TSA-II and the Timed protocol is explained by the most sensitive anchor for each scale lying at opposing ends to one another. That is, an individual who exhibits high sensitivity to cold sensation will report CPT at higher temperatures on the TSA-II and at a shorter time period during the Timed protocol.

Previously, it was thought that diverse testing methodologies applied across QST studies made comparative interpretation of results difficult [14]. The results of this study support this statement; as association between different measurement protocols and the reference standard is only moderate at best. Notwithstanding this, the results of this study support the use of ice to measure CPT in a clinical setting, with the PVAS protocol being the most reliable of the two clinical protocols, but the Timed protocol more closely associated with the reference standard.

Previous studies have utilised a clinical measure of CPT using ice [11, 15] using various methodologies. Maxwell and Sterling [11] applied a protocol of ice to the skin for 10 seconds with a numerical rating scale for pain (NRS, similar to our PVAS protocol), whereas Rebbeck et al. [15] applied ice to the skin for only 5 seconds and obtained similar NRS for pain. Both of these studies were in participants with neck pain (whiplash associated disorder and chronic neck pain), with Rebbeck et al. [15] comparing their neck pain group to a control group, with neither study reporting reliability statistics. To our knowledge, there are no previous studies investigating the reliability of the PVAS/NRS clinical protocol for CPT.

We used 30 seconds for the PVAS protocol instead of the 5 or 10 seconds previously used, as our pilot testing revealed minimal number of participants reported any pain at less than this 30-second time period. Our group consisted of healthy younger individuals (mean age 22.6 (SD 1.81) years versus 41 (11.2) [15], and 43.3 (SD 12.7) years [11]). It is well documented [8, 16] that sensory thresholds increase with age and as such; our younger cohort may have required longer exposure to the cold stimulus before reporting any discomfort or pain associated with the stimulus. Furthermore, results from Rebbeck et al. [15] indicate that they experienced a floor effect in their clinical measurement, as they reported a mean of 0.0/10 on the NRS for both the neck pain and control groups across all anatomical sites except the neck (mean 2.0/10 for the neck pain group). The descriptive data was not reported in Maxwell and Sterling [11], so comparisons could not be made.

Two previous studies reported reliability statistics for the Timed ice protocol to the temples and wrists in healthy participants, with a cut-off limit of 180 sec [10, 17]. One study reported reliability coefficients of 0.63 and 0.91 for left and right temples, respectively, and a mean time to pain threshold of 24 sec [17], while the other study reported ICCs of 0.75 to 0.92 between sessions and a mean time to discomfort of 20.5 sec [10]. Our findings are consistent with this previous work, with an overall mean time to pain of 21.5 sec.

The effectiveness of both the TSA-II protocol and the Timed Ice protocol is dependent upon the ability and awareness of each participant to comprehend their CPT and accurately report this, either via pushing a button or saying “stop.” The perception of discomfort/pain was variable between sessions. Focusing on the personal perception of pain and discomfort may have been a foreign concept to a young, healthy population such as this. It has been shown that QST is vulnerable to bias related to the attention, motivation, and cognitive processing ability of participants [18]. In contrast, the PVAS ice protocol involved ice on the skin for a set duration (30 seconds) and did not require the participant's input to end the test, unlike the “open-ended” nature of the TSA-II and Timed protocols. The challenge of identifying an end-point to the test for individual participants may explain the increased variation in response for these 2 protocols.

Another explanation for the stronger repeatability between sessions seen with the PVAS protocol is that participants may have remembered their PVAS rating between sessions for the PVAS protocol but were blinded to the CPT temperature and time recorded in the TSA-II and Timed protocols. As such, it is possible that the increased reliability in the PVAS protocol is due to memory recall of the participants, with no such bias present in the other two protocols.

Although the time of day and instructions were standardised and the same assessors performed all tests in this study, there was still an obvious lack of reliability between sessions of the TSA-II and Timed protocols. No familiarisation was undertaken in the TSA-II protocol. It has previously been recommended that participants should be familiarised with the application of the test stimuli in nonpainful ranges before baseline measurements are taken, to avoid potential anxiety associated with testing [10]. Others recommend training participants over two to four sessions prior to CPT testing so that their individual pain thresholds vary by no more than 0.8°C between the last two test sessions and that participants be excluded from the study if they fail to perform in a consistent manner during the trial sessions [19]. In future studies, greater participant familiarisation prior to testing may improve reliability measures.

All three protocols consistently showed significant differences in the CPT between anatomical locations. This is consistent with previous research [8, 10], and it is advised that when comparing CPT results with normative data, specific anatomical locations should be matched. We chose to test CPT across six anatomical locations (Table 1) in healthy participants. It is recommended that, in patients with painful disorders, if the purpose is to determine the severity of sensory dysfunction, QST should generally been performed in the area of maximal pain [20]. However, this can be problematic as QST data is highly location specific and existing normative data is currently limited to only a small number of anatomical locations, such as face, hands, and feet [8, 21]. To our knowledge, this is the first study to report normative data for the other anatomical locations that have been used in our study.

We chose to only investigate cold as a sensory measure of pain threshold in this study. Assessment of response to multiple sensory stimuli including thermal and mechanical is recommended when quantifying sensory processes [8], as pain is multidimensional in nature and one modality alone is not sufficient to fully characterise an individual's pain profile or the complexity of pain perception.

## 8. Conclusion

The PVAS ice protocol may be more reliable for determination of CPT in a clinical setting, but the Timed protocol may be more valid when compared to a reference standard. Both protocols require further testing in pain populations in order to determine the optimal exposure time to the cold stimulus to ensure maximum reliability and validity of the measure.

## Figures and Tables

**Table 1:** Anatomical locations for the three protocols.

Anatomical site	Location
Neck	2 cm lateral to the spinous process of C7
Shoulder	5 cm distal to the anterior border of the acromion
Elbow	Lateral epicondyle of the humerus
Thigh	5 cm distal to the pubic crease on the adductor longus tendon
Knee	Inferior pole of the patella at the insertion of the patella tendon
Ankle	Medial aspect of the Achilles tendon, level with the medial malleolus of the tibia

**Table 2:** Mean ± standard deviation (SD), intraclass correlation coefficient (ICC), and limits of agreement (LoA) between sessions for each protocol.

Location	Session 1	Session 2	ICC (95% CI)	95% LoA
	Mean ± SD			
TSA-II°C				

Neck	19.2 ± 7.1	16.0 ± 8.0	0.679 (0.370 to 0.853)	−8.7 to 15.0
Shoulder	15.2 ± 7.8	14.9 ± 7.3	0.396 (−0.021 to 0.695)	−16.0 to 16.5
Elbow	11.5 ± 8.7	12.4 ± 7.3	0.555 (0.184 to 0.788)	−15.8 to 13.9
Thigh	13.6 ± 6.9	13.7 ± 7.5	0.185 (−0.247 to 0.556)	−18.2 to 18.0
Knee	8.1 ± 5.8	6.7 ± 6.9	0.492 (0.099 to 0.752)	−11.2 to 14.0
Ankle	9.5 ± 7.3	8.9 ± 7.0	0.390 (−0.027 to 0.692)	−15.1 to 16.2

Total			0.545 (0.414 to 0.655)	−15.7 to 14.2

Timed protocol sec				

Neck	12.8 ± 7.4	15.3 ± 6.4	0.483 (0.088 to 0.747)	−11.5 to 16.2
Shoulder	15.5 ± 7.3	16.6 ± 7.0	0.590 (0.234 to 0.807)	−11.2 to 13.6
Elbow	19.7 ± 14.6	24.6 ± 15.1	0.868 (0.710 to 0.943)	−10.0 to 19.6
Thigh	24.1 ± 14.4	26.3 ± 15.2	0.708 (0.416 to (0.867)	−20.0 to 24.5
Knee	27.8 ± 12.9	34.1 ± 17.8	0.517 (0.132 to 0.766)	−23.5 to 35.8
Ankle	18.8 ± 12.4	21.6 ± 13.0	0.597 (0.244 to 0.810)	−19.5 to 25.1

Total			0.714 (0.619 to 0.788)	−23.3 to 16.8

PVAS protocol 0–100 mm				

Neck	29.5 ± 19.1	28.2 ± 21.5	0.870 (0.714 to 0.944)	−18.7 to 23.2
Shoulder	20.9 ± 13.8	17.3 ± 10.7	0.578 (0.216 to 0.800)	−19.8 to 26.0
Elbow	15.9 ± 13.3	16.4 ± 13.6	0.569 (0.203 to 0.795)	−20.1 to 20.2
Thigh	13.2 ± 17.0	13.2 ± 13.2	0.733 (0.459 to 0.880)	−20.5 to 19.5
Knee	9.5 ± 10.5	9.5 ± 11.7	0.653 (0.329 to 0.840)	−16.8 to 15.0
Ankle	16.4 ± 15.9	20.9 ± 18.8	0.752 (0.492 to 0.889)	−25.4 to 16.8

Total			0.783 (0.706 to 0.841)	−21.0 to 20.4

**Table 3:** Mean ± standard deviation (SD) and ICC (95% confidence intervals) between protocols.

Location	TSA-II°C	Ice protocols		TSA-II and PVAS	TSA-II and time
		Timed sec	PVAS mm	ICC (95% CI)	
Neck	17.6 ± 6.9	14.1 ± 5.9	28.9 ± 1.9	−0.013 (−0.424 to 0.403)	−0.530 (−0.774 to −0.150)
Shoulder	15.1 ± 6.3^1^	16.1 ± 6.4	19.1 ± 10.9^1^	0.040 (−0.380 to 0.446)	−0.254 (−0.604 to 0.178)
Elbow	12.0 ± 7.1^1,2^	22.2 ± 14.3^1,2^	16.1 ± 11.9^1^	0.182 (−0.301 to 0.514)	−0.525 (−0.771 to −0.143)
Thigh	13.6 ± 5.6^1,5,6^	25.2 ± 13.7^1,2^	13.2 ± 14.2^1,2^	0.190 (−0.243 to 0.559)	−0.516 (−0.766 to −0.131)
Knee	7.3 ± 5.5^1,2,3^	30.9 ± 13.6^1,2,3,4^	9.5 ± 10.1^1,2,3^	0.214 (−0.219 to 0.576)	−0.297 (−0.633 to 0.133)
Ankle	9.2 ± 6.0^1,2^	20.2 ± 11.4^1,5^	18.6 ± 16.2^1,5^	0.153 (−0.278 to 0.532)	−0.496 (−0.754 to −0.104)

^1^Significantly different to neck; ^2^significantly different to shoulder; ^3^significantly different to elbow; ^4^significantly different to thigh; ^5^significantly different to knee; ^6^significantly different to ankle.
